# Interposition arthroplasty in post-traumatic temporomandibular joint ankylosis: A retrospective study

**DOI:** 10.4103/0970-0358.59277

**Published:** 2009

**Authors:** Satyaswarup Tripathy, Mohd Yaseen, Nitya N. Singh, L. M. Bariar

**Affiliations:** Department of Plastic Surgery, J.N. Medical College, AMU, Aligarh, UP, India

**Keywords:** Interpositional arthroplasty, temporal fascia, temporomandibular joint ankylosis

## Abstract

Temporomandibular joint ankylosis which is most frequently caused by trauma, presents with restriction in mouth opening in early stages and if children are the victim and not treated early, it presents with growth retardation of the involved mandibular side. Various methods are available for surgical correction. We have reviewed our experience with the efficacy of different interpositional materials in post-traumatic cases in our set up with special reference to temporal fascia over last three years. Twenty seven patients with history of trauma, mostly fall from height, have been studied. They were evaluated clinically and by computed tomography (CT) scan, orthopantogram and x- ray lateral oblique view. The most common age group was 10-15 years with mean 12.5 years and male to female ratio 1:2. Preoperative mouth opening (inter incisor distance) was 1-2 mm in 17 cases and 2-4 mm in 10 cases. We have used temporalis fascia in nine, costochondral graft in seven, silastic sheets in five and T-plates in six cases. Post-operatively, adequate mouth opening of 30-50 mm was observed in six months follow-up and more than 50 mm at one year follow up in 21 cases out of which nine cases have interpositional material as temporalis fascia alone. The postoperative period was uneventful in all cases and none required re-operation for recurrences. We conclude that interpositional arthroplasty, especially with pedicled temporal fascia, is the best method to prevent recurrences and establish good mouth opening and full range of jaw movements.

## INTRODUCTION

Temporomandibular joint (TMJ) ankylosis is a disorder wherein a stiff joint leads to to restriction of mouth opening. It is due to fibrous or bony union between the head of condyle of mandible and the glenoid fossa of temporal bone. It may be caused by trauma, infection, degenerative joint disease, prolonged mandibulo-maxillary fixation, prior surgery, inflammatory conditions like rheumatoid arthritis, systemic diseases (ankylosing spondylitis, scleroderma) and polyarthritis.

Most common amongst them include post traumatic cases (13 to 100%).[1] It is classified into true or false ankylosis by Kazanjian.[2] In true ankylosis, there is bony or fibrous adhesion between surfaces of joint within joint capsule; whereas in false ankylosis, the pathology lies in surrounding structures. It is one of the most overlooked and undermanaged condition in the initial management of trauma and results in disability and subsequent morbidity.

The unmanaged temporomandibular joint ankylosis leads to restricted mouth opening, impairment of mastication, poor oral hygiene, caries tooth, speech disturbances, bird face deformity, facial asymmetry aggravating psychological stress, growth disturbances in maxilla and mandible, especially in children and bilateral cases (mandibular micrognathia) and airway compromise. Therefore, early diagnosis and surgical intervention are important.

The aim of treatment is to establish joint movement with adequate mouth opening and to prevent recurrences. Though various techniques have been described, recurrence is a major problem in treating this condition.

### Surgical options include:

- Gap Arthroplasty (resection of bony mass without interposition material)
- Joint Reconstruction (by bone grafts or microvascular reconstruction by transfer of second metatarsophalangeal joint,[3] distraction osteogenesis,[4] or joint prosthesis).
- Interpositional Arthroplasty(resection of bony mass with interposition of biological or non biological material)

Several materials have been used for interpositional arthroplasty- temporal fascia, temporalis or masseter muscle, fascia lata, dermis, full thickness skin, autologous costochondral cartilage. Non-biological options comprise insertion of silastic materials and T-plates insertion among the non biological material options.

The present study describes our experience with the efficacy of various interpositional materials in post traumatic temporomandibular joint ankylosis in our set up over the last three years period.

## MATERIAL AND METHODS

This is a retrospective study carried out in the Department of Plastic Surgery, J. N. Medical College and Hospital, Aligarh Muslim University, Aligarh. The period of study was December 2004 to November 2007. All cases had neglected, overlooked, post traumatic temporomandibular joint ankylosis. All patients were assessed by clinical examination for restricted mouth opening. Protrusive and lateral movements were assessed in all patients. Inter incisor distance was recorded by using vernier caliper. All patients underwent X-Ray skull lateral oblique view, orthopantomogram (OPG) and computed tomography. Operative techniques were selected from the available case records.

### Operative Technique

All patients were operated under general anaesthesia with fibre-optic nasotracheal intubation. An inverted hockey stick incision, which follows the natural crease in front of the tragus with temporal extension was used. On reaching the temporo-parietal fascial layer, the superficial temporal artery and vein, which run just above the surface of the fascial layer, and the branches of the facial nerve, which run deep to it, just above the periosteum over the zygomatic arch were identified.

Above the zygomatic arch the superficial layer of the temporal fascia was incised in an oblique line running from the tragus to the superior temporal line. This incision was parallel to the inverted hockey-stick incision. A mosquito haemostat was used to dissect bluntly along the external auditory canal in an anterior-medial direction to the level of the temporomandibular joint capsule. A number 15 blade was used to make an incision along the root of the zygoma through the superficial temporal fascia and the periosteum, contiguous with the incision superior to the arch. With blunt haemostat dissection, a plane was developed through this incision, just above the white, glistening temporomandibular joint capsule. While elevating this “pocket,” we used a blade to extend the fascial release to the most inferior part of the tragus. This technique allowed us to retract the superficial temporal vessels anteriorly and preserve this vital pedicle of our temporal fascial flap. Approximately one cm of bony mass causing ankylosis was removed using osteotome. A trial mouth opening was attempted and any fibrous tissue causing restriction of the mouth opening was removed. Interpositional arthroplasty was done using pedicled temporal fascia [Figure 1d], costochondral graft, silastic sheet and T-plates [Figure 2c]. Haemostasis was secured and wound closed after noting intraoperative passive mouth opening.

**Figure 1d:**
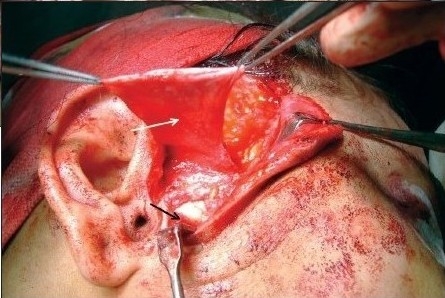
Intraoperative photograph showing raised temporalis fascia (white arrow) and showing the gap (black arrow)

**Figure 2c:**
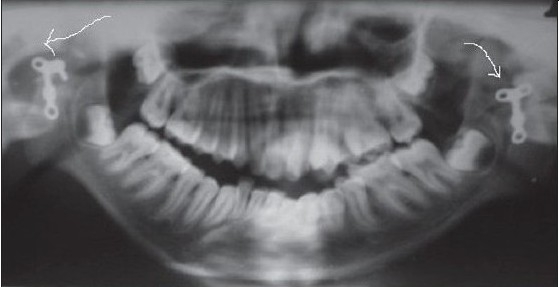
Postoperative orthopantomogram showing bilateral interposition of T-plates (arrow)

**Figure 3:**
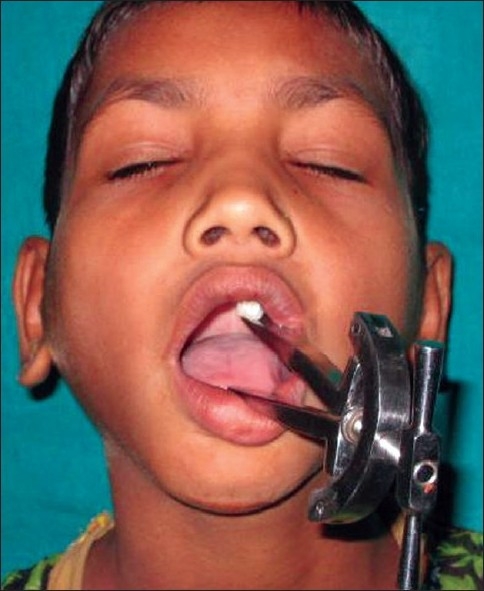
Postoperative frontal view showing adequate mouth opening and use of Heister's mouth exerciser

Postoperative mouth opening exercises were initiated on the third postoperative day and later, both active and passive exercises were allowed, the latter using Heister mouth prop [Figure 3] till six months to one year. Pre and postoperative mouth opening were measured by inter incisor distance and compared. Patients were followed up at one, three and six months and one year interval.

## RESULTS

We studied 27 patients, of whom, 24 had bony ankylosis [Figure 1c, 2b] and three had severe fibrous ankylosis. Despite three to six months of active and passive mouth opening exercises, the latter group of three showed no improvement. Therefore, interposition arthroplasty was done in all patients using various methods. Nine cases were treated by pedicled temporal fascia, seven by costochondral graft, six by T-plates and five cases by silastic sheets as an interposition material on a random basis. Interestingly, females were twice more frequently afflicted by TMJ ankylosis than males in our study. The age ranged from 4 to 20 years with the most common group falling in 10-15 years age group [Table 1]. In most of the cases history of trauma was more than four years. All cases were post traumatic and in the majority, a history of fall from a height was elicited.

**Figure 1c:**
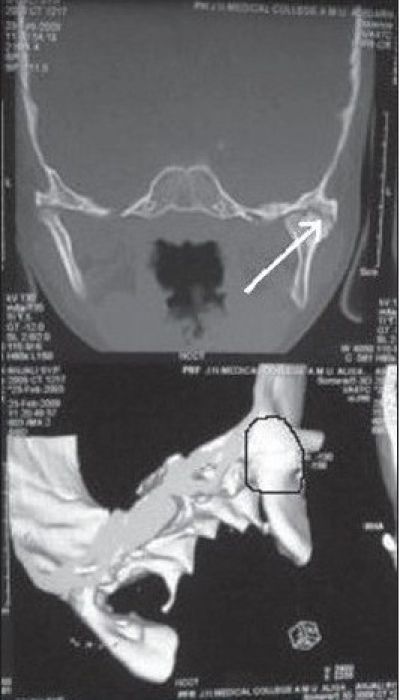
Preoperative C T scan showing right sided temporomandibular joint ankylosis in both coronal (arrow) and 3D reconstruction (circle)views

**Figure 1e:**
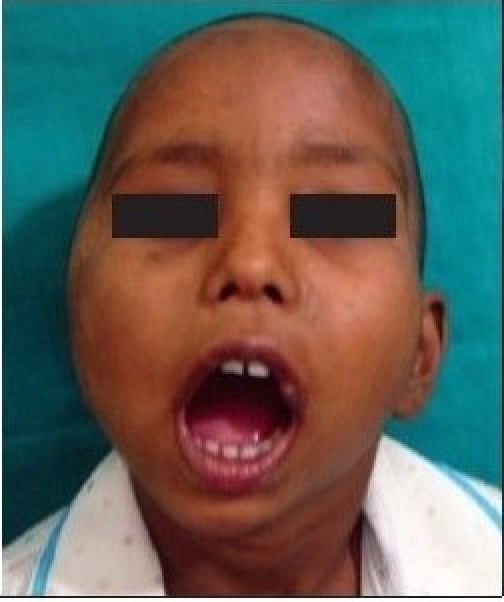
Postoperative frontal view showing adequate mouth opening

**Figure 1f:**
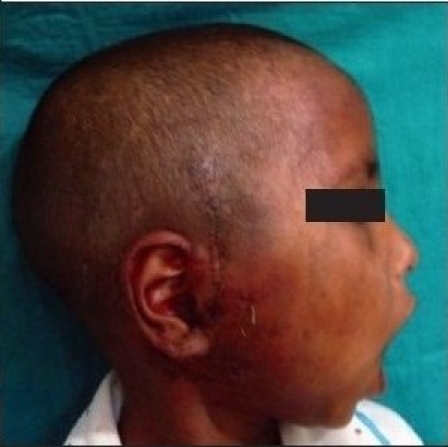
Postoperative lateral view showing adequate mouth opening

**Figure 2a:**
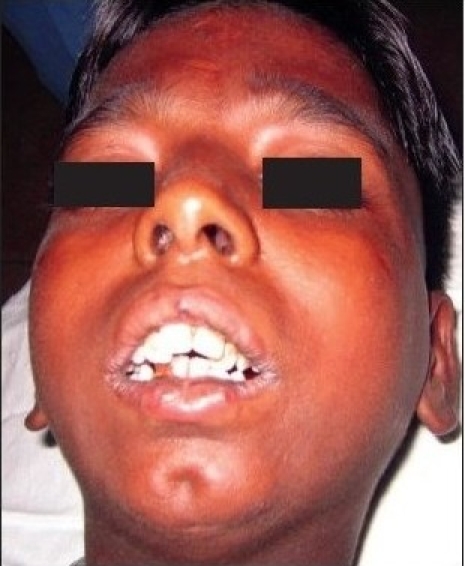
Preoperative photograph showing decreased mouth opening due to bilateral temporomandibular joint ankylosis

**Figure 2b:**
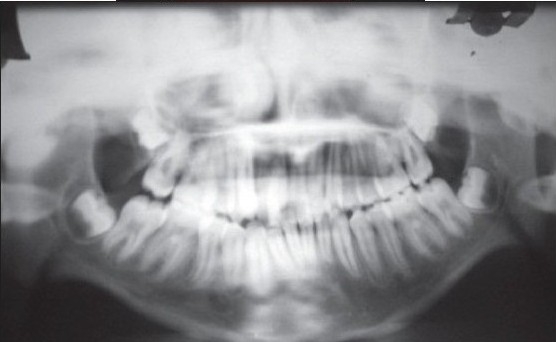
Preoperative orthopantomogram showing bilateral temporomandibular joint ankylosis

**Table 1:** Patients' details

*Age group*	*Male*	*Female*	*Total no of cases*	*%*
0 - 5	1	1	2	7.4
5 - 10	2	4	6	22.2
10 - 15	4	10	14	51.8
15-20	2	3	5	18.5

Pre operative mouth opening (inter incisor distance) was 1-2 mm in 17 patients and 2-4 mm in ten patients [Table 2]. Lateral and protrusive movements were severely restricted and were not assessable in all patients preoperatively. Postoperative satisfaction and adequate mouth opening achieved was 30-50 mm in almost all cases in six months and more than 50 mm in 21 cases in one year follow-up period [Table 2] out of which nine cases had temporal fascial flap as the interpositional material [Figure 1a–f], five had costochondral graft, four had T-plates [Figure 2–d] and there were three cases with silastic sheets [Table 3]. The postoperative period was uneventful in all cases. None of the cases was reported with recurrences.

**Table 2:** Pre and postoperative mouth opening

*Inter Incisor Dist. (Mm)*	*Pre Op (No of Pts.)*	*Post Op (No of Pts)*	*Follow up at (M And Y)*	*%*
0 - 2	17	-	-	63
2 - 5	10	-	-	37
5 - 10	-	-	-	-
10 - 30	-	-	-	-
30 - 50	-	25	6 month	92.6
> 50	-	21	1 year	78

**Figure 1a:**
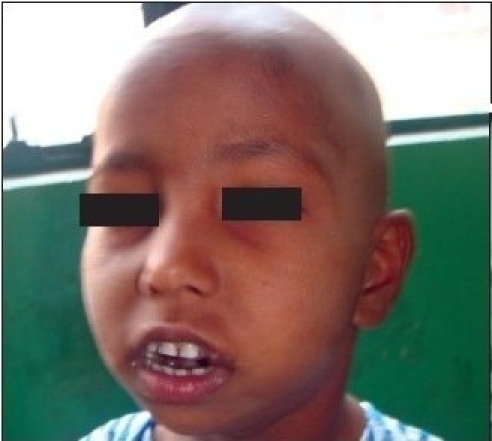
Preoperative photograph showing decreased mouth opening due to right sided temporomandibular joint ankylosis

**Figure 1b:**
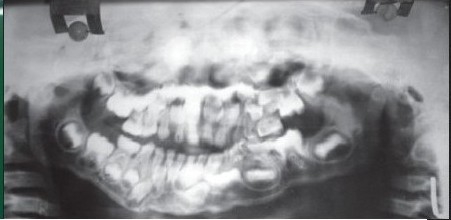
Preoperative orthopantomogram showing right sided temporomandibular joint ankylosis

**Figure 2d:**
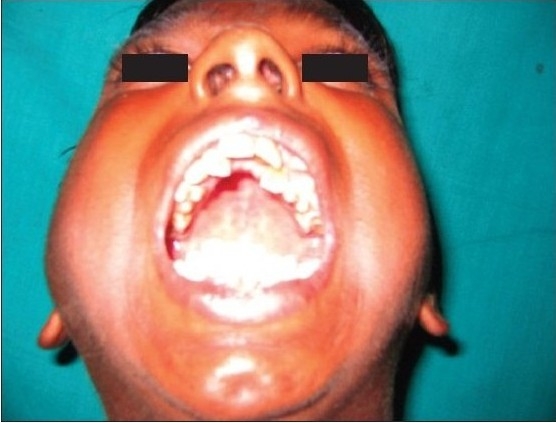
Postoperative frontal view showing adequate mouth opening

**Table 3:** Type of operation

*Interposition Material*	*No of cases*	*>50Mm Mouth Opening At 1 Yr*	*Success rate %*
Temporal fascia (pedicled)	9	9	100
Costochondral Graft	7	5	71
T - plates	6	4	66
Silastic sheet	5	3	60

## DISCUSSION

Normal mouth opening in adults is between 40-56 mm. This distance varies in children depending upon the age and stature of the child. Temporomandibular joint ankylosis not only affects mouth opening but also the normal growth pattern of the mandible.

Temporomandibular joint ankylosis can be due to fibrous or osseous union within the temporomandibular joint, causing a firm restriction of the condyle, which is generally not associated with pain. Digital palpation of the ankylosed temporomandibular joint during maximal movements demonstrates none or very limited translation of the condyle.[5–7] Limited mouth opening due to ankylosis does not improve by conservative therapy. Topazian reviewed gap arthroplasty without interposition and reported a recurrence rate of 53%.[1]

The zone of excision should be sufficiently wide to prevent ankylosis but should produce little change in the vertical height of mandible. Since prevention of reankylosis is difficult, Rajgopal and associates,[8] have suggested radical condyle and neck removal as well as coronoidectomy. Of course, the vertical ramus height becomes greatly reduced.

Most authors tend to agree, however, that recurrent ankylosis is less likely if material is interposed between the divided bone ends. Controversy, however, arises over whether to place alloplastic material (Proplast, Teflon, Silastic methyl methacrylate etc.) or autogenous tissues (fascia lata, muscle, full thickness skin or cartilage) into the defect.[9] We used temporal fascia (pedicled), costochondral graft, silastic sheet and T-plate as an interposition material. We found almost no recurrences after using pedicled temporal fascia as interpositional material in one year follow-up.

All patients under treatment showed distinct improvement, both in their articular function and symptoms, in the study of Valentini *et al.*[10] while using various methods of interpositional arthroplasty. The use of silastic as alloplastic material could be associated with an increased persistence of local symptoms and a higher risk of foreign body granuloma. This may favour ankylosis and relapse and hinder rehabilitation.[6] Smith *et al*, have reported erosion of Teflon proplast implant into middle cranial fossa.[11]

Biological materials that have been used have the problems of degeneration and fibrosis with time, resulting in reankylosis. Demir *et al*. found no recurrence in preserved costochondral homograft as an interposition material. The dependable supply of optimally preserved homologous costal cartilage is not possible at all set ups.[12] Poswillo concluded that whereas costochondral transplants adapt to the growth requirement of the surrounding tissue,[13] but costochondral grafts require another operative site with its attendant complications and poor patient compliance.

Abul Hassan[14] concluded that deep temporal fascia is supplied by middle temporal artery, a constant branch of superficial temporal artery. Temporalis fascia flap is a locally available axial pattern flap, easy to elevate and available in all clinical situations. This vascularized flap has fewer chances of subsequent absorption and fibrosis[15] Although our study was small, the results were in favour of temporal fascia as an interposition material. Hence, in our opinion, it is the best method to prevent recurrence of temporomandibular joint ankylosis.

## CONCLUSION

The main goal of treating temporomandibular joint ankylosis is to achieve adequate mouth opening with minimal chance of recurrence in long term follow up. In this retrospective study, we selected the patients undergoing interposition arthroplasty using various methods. Results have shown that there was adequate mouth opening with no recurrence in patients in whom temporalis fascia was used as an interpositional material at one year follow-up. As treatment of recurrence is not only difficult but also has a poor prognosis, we conclude that interposition arthroplasty, especially with temporal fascial flap interposition is a good option to prevent recurrence in our set up.
